# Ultrashort-Echo-Time MRI of the Disco-Vertebral Junction: Modulation of Image Contrast via Echo Subtraction and Echo Times

**DOI:** 10.3390/s24175842

**Published:** 2024-09-09

**Authors:** Karen C. Chen, Palanan Siriwananrangsun, Won C. Bae

**Affiliations:** 1 Department of Radiology, VA San Diego Healthcare System, San Diego, CA 92161, USA; karenchanchen@gmail.com; 2 Department of Radiology, Siriraj Hospital, Bangkok 10700, Thailand; palanan.siri@gmail.com; 3 Department of Radiology, University of California-San Diego, La Jolla, CA 92093, USA

**Keywords:** low back pain, disco-vertebral junction, cartilage endplate, lumbar spine, disc degeneration, ultrashort echo time, MRI

## Abstract

Introduction: The disco-vertebral junction (DVJ) of the lumbar spine contains thin structures with short T2 values, including the cartilaginous endplate (CEP) sandwiched between the bony vertebral endplate (VEP) and the nucleus pulposus (NP). We previously demonstrated that ultrashort-echo-time (UTE) MRI, compared to conventional MRI, is able to depict the tissues at the DVJ with improved contrast. In this study, we sought to further optimize UTE MRI by characterizing the contrast-to-noise ratio (CNR) of these tissues when either single echo or echo subtraction images are used and with varying echo times (TEs). Methods: In four cadaveric lumbar spines, we acquired 3D Cones (a UTE sequence) images at varying TEs from 0.032 ms to 16 ms. Additionally, spin echo T1- and T2-weighted images were acquired. The CNRs of CEP-NP and CEP-VEP were measured in all source images and 3D Cones echo subtraction images. Results: In the spin echo images, it was challenging to distinguish the CEP from the VEP, as both had low signal intensity. However, the 3D Cones source images at the shortest TE of 0.032 ms provided an excellent contrast between the CEP and the VEP. As the TE increased, the contrast decreased in the source images. In contrast, the 3D Cones echo subtraction images showed increasing CNR values as the second TE increased, reaching statistical significance when the second TE was above 10 ms (*p* < 0.05). Conclusions: Our study highlights the feasibility of incorporating UTE MRI for the evaluation of the DVJ and its advantages over conventional spin echo sequences for improving the contrast between the CEP and adjacent tissues. Additionally, modulation of the contrast for the target tissues can be achieved using either source images or subtraction images, as well as by varying the echo times.

## 1. Introduction

The disco-vertebral junction (DVJ) of the human lumbar spine is a complex structure that includes several layers of tissues. Among these, the cartilaginous endplate (CEP) is sandwiched between the bony vertebral endplate (VEP) and the nucleus pulposus (NP) of the intervertebral disc (IVD) [1]. The CEP is a thin ~1 mm layer of connective tissue [2] whose roles include mechanical stabilization as well as providing a pathway for nutrient transport from the vertebral body via vascular canals [2,3,4]. Unlike the natural history of IVD degeneration, which has been studied extensively [5], how the CEP changes with aging and with low back pain is only beginning to be studied.

**Figure 1:**
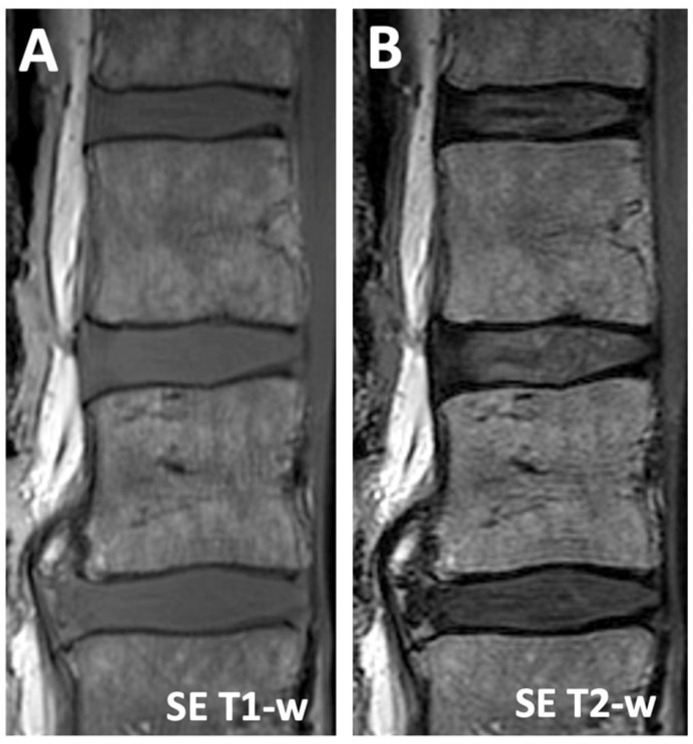
Conventional sagittal MR imaging of the human lumbar spine performed with (**A**) spin echo T1-weighted and (**B**) spin echo T2-weighted sequences. The cartilage endplate at the disco-vertebral junction (interface between vertebral body and intervertebral disc) has low signal intensity and is indistinguishable from the vertebral endplate, the cortical bone of the vertebral body.

The CEP and the VEP are seen with low contrast using the conventional sagittal magnetic resonance imaging (MRI) protocol including spin echo T1- and T2-weighted sequences (Figure 1). This is mainly due to the intrinsically low T2 properties of these tissues, which decay to a low signal intensity when the echo time of the sequence is not sufficiently short. Ultrashort-echo-time (UTE) MR imaging overcomes this limitation, providing means to acquire sufficient signal from short-T2 tissues [6,7,8,9,10,11,12,13]. Furthermore, UTE acquisition at multiple echo times (TEs) and echo subtraction image processing accentuate short-T2 tissues [14,15,16,17]. In this way, we previously showed that a normal CEP could be depicted with a continuous, linear, and high signal intensity on the subtraction image [16], in agreement with the anatomy [18]. In cadaveric human spines, the normal and predominant UTE MR morphology of the CEP in the sagittal plane is that of a structure with a continuous, linear, and high signal intensity [17], as one would expect from the anatomy [18].

The rationale for the echo subtraction technique with UTE MRI is simple: the first echo image, acquired with the ultrashort echo time contains signal intensity from both short- and long-T2 tissues, while the source image from later TEs will contain only the longer-T2 components, whose high signal intensity has decayed relatively little over this duration. The subtraction image will therefore filter out the long-T2 components and accentuate the short-T2 tissues. While this method is simple and effective, no formal inquiry into how to best optimize the TE spacing to achieve the best contrast between different spinal tissues has been performed.

The goal of this study was to determine the UTE MRI image contrast of the DVJ when the TE spacing between the first and the second echo was varied. This study provides insight into setting the scan parameters for UTE MRI of the lumbar spine when modulation of specific tissue contrast (e.g., to distinguish the CEP from the IVD) is desired.

## 2. Materials and Methods

This study did not involve human subjects or live animals and was exempt from approval by the institutional review board.

### 2.1. Samples

Cadaveric human lumbar spines containing L1 to S vertebrae were obtained from 4 donors (3 females, 1 male, aged 42 to 79 years). The spines were harvested *en bloc* from the cadavers and frozen at −70 °C until scanning. The spines were slowly thawed a day prior to scanning in a cooler, transported to the MRI facility, and imaged.

### 2.2. MR Imaging

The spines were imaged on a 3-Tesla scanner (DV750, General Electric Healthcare, Milwaukee, MI, USA) using a clinical 8-channel transmit/receive knee coil. The knee coil was appropriately sized for the specimens, which measured approximately 20 cm in length from L1 to S1. We positioned the specimens in a head-first prone orientation, ensuring the vertebral bodies were near the center of the coil. The field of view was adjusted to include the region from the lower part of L1 to the upper part of L5, aligning with the coil’s coverage area.

All samples were imaged through the mid-sagittal plane using the following protocols: (1) T1-weighted spin echo sequence (SE T1-w) consisting of repetition time TR = 650 ms, echo time TE = 10 ms, field of view FOV = 20 cm, phase encoding × read out = 384 × 384, and slice thickness = 2.5 mm; (2) T2-weighted spin echo sequence (SE T2-w) consisting of TR = 3700 ms, TE = 100 ms, and other parameters similar to those of the T1-weighted sequence; (3) 3D Cones sequence (a UTE MRI sequence) consisting of TR = 47 ms, 10 TEs = 0.032, 1, 2.5, 4.5, 6.7, 9, 11, 13, 14, and 16 ms (acquired over 4 separate scans using the same receiver gain), number of spokes = 6000, image matrix = 384 × 384, flip angle = 19 deg, slice thickness = 2 mm, and number of slices = 28.

### 2.3. MRI Image Processing

**Figure 2:**
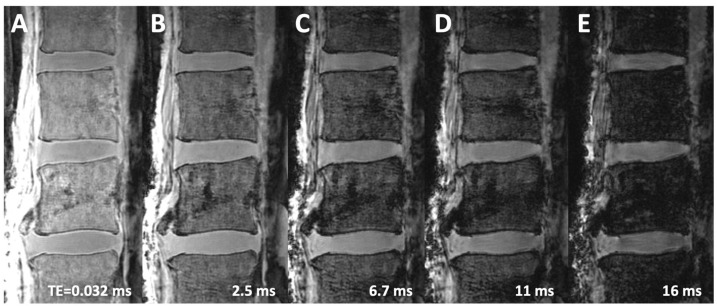
3D Cones (an ultrashort-echo-time, UTE, sequence) images acquired at echo times (TEs) of (**A**) 0.032 ms, (**B**) 2.5 ms, (**C**) 6.7 ms, (**D**) 11 ms, and (**E**) 16 ms. The cartilage endplate at the disco-vertebral junction has a high signal intensity in (**A**), appearing distinct from the adjacent vertebral endplate (VEP) and the intervertebral disc. In later TE source images (**B**–**E**), the CEP becomes progressively darker and indistinguishable from the VEP.

**Figure 3:**
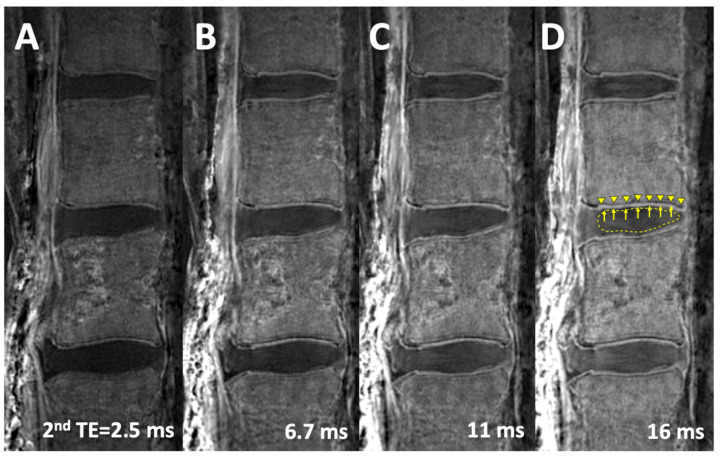
3D Cones subtraction images, acquired by digitally subtracting the 2nd echo image (at various TEs) from the 1st echo image at TE = 0.032 ms. Subtraction images using the 2nd TE of (**A**) 2.5 ms, (**B**) 6.7 ms, (**C**) 11 ms, and (**D**) 16 ms. By increasing the 2nd TE, visual improvement in the contrast between the cartilage endplate (arrows) and the adjacent vertebral endplate (arrowheads) and nucleus pulposus (dotted line) can be seen.

Using the 3D Cones UTE MR images acquired at different TEs, echo subtraction processing was conducted. The second echo image (Figure 2B–E) was digitally subtracted from the first echo image acquired at the shortest TE of 0.032 ms (Figure 2A), creating an image that highlighted the short-T2 signal from the CEP (Figure 3). In Figure 3C, the normal CEP (arrows) is clearly visible at a continuous, linear, and high signal intensity, easily distinguished from the adjacent NP (dotted line) and bony vertebral endplate (arrowheads). In contrast, the conventional spin echo images (Figure 1A,B) show both the CEP and the VEP with low signal intensity, making it difficult to differentiate them.

### 2.4. Signal-to-Noise Ratio (SNR) and Contrast-to-Noise Ratio (CNR)

**Figure 4:**
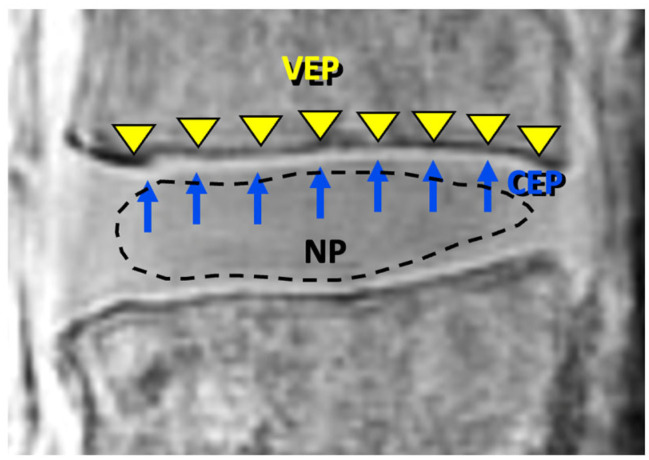
Regions of interest for the bony vertebral endplate (VEP), cartilage endplate (CEP), and nucleus pulposus (NP), indicated with arrowheads, arrows, and dotted line, respectively. For each specimen, the average signal intensity within each ROI was determined.

On both source images and subtraction UTE images, regions of interest (ROIs) were placed on the mid-sagittal slice showing the largest cross section of the IVD. The ROIs representing the bony vertebral endplate (VEP), the cartilage endplate (CEP), and the nucleus pulposus (NP) tissues were placed manually (Figure 4) by a scientist with over 10 years of experience in imaging research. For each spine and for each tissue (VEP, CEP, NP), multiple ROIs were placed from L2 to L4 and averaged to determine the mean signal intensity (*SI_mean_*) for the given tissue. ROIs were also placed in the background (air) to determine the standard deviation (*SI_SD_*) of the noise signal intensity.

The ROIs for the VEP and CEP were placed only in the areas demonstrating “normal” morphology, i.e., in areas with continuous, linear, and high signal intensity near the DVJ. A few focal areas that exhibited “abnormal” morphology (any deviation from the normal one, including obvious signal loss, thickening, or irregularity [17]) were excluded from the analysis. For quality control, we initially evaluated the slice-to-slice variability in the signal intensity as a coefficient across five consecutive slices of two datasets and found less than 2% variability for the first echo UTE images.

The SNR for a given tissue was determined as the mean signal intensity divided by the standard deviation of the noise in the air [19] (Equation (1)).

(1) $$SNR^{tissue}=\frac{SI_{mean}^{tissue}}{SI_{SD}^{noise}}$$

The CNR was determined as the difference in mean signal intensity between selected anatomies divided by the standard deviation of the noise [20] (Equation (2)).

(2) $$CNR^{tissue1-tissue2}=\frac{SI_{mean}^{tissue1}-SI_{mean}^{tissue2}}{SI_{SD}^{noise}}$$

The SNR values were determined for CEP, NP, and VEP, while the CNR values were determined between the adjacent anatomies of the CEP and the NP and between the CEP and the VEP.

### 2.5. Statistics

The effect of the sequence (SE T1, SE T2, and 3D Cones at TE = 0.032 ms) on the CNR values were compared using ANOVA [21,22] with Tukey post hoc test. For 3D Cones source images, the effect of the TE on the CNR values was determined using repeated-measures ANOVA. For 3D Cones echo subtraction images, the effect of the 2nd echo TE on the CNR values was also assessed using repeated-measures ANOVA. Statistical analyses were performed using Systat software (v12, Grafiti LLC, Palo Alto, CA, USA). The significance level was set at 5%. The power of the test (1-*β* error probability) was determined from the effect size for each factor, using partial eta-squared values in each ANOVA analysis and G*Power software (Version 3.1.9.6) [23,24].

## 3. Results

In the spin echo T1-w and T2-w images (Figure 1), it was difficult to distinguish the CEP from the VEP, as both tissues had similarly low signal intensity. In the 3D Cones source images (Figure 2), the image contrast heavily depended on the TE: visually, at the shortest TE of 0.032 ms, there was the best contrast between the CEP and the VEP, as well as between the CEP and the NP (i.e., all three tissues were distinguishable). As the TE increased, the contrast between these three tissues decreased, and the tissues became less distinct. In the 3D Cones subtraction images (Figure 3), the image contrast between the DVJ tissues also depended on the TE of the subtraction images, with the three tissues near the DVJ (NP, CEP, VEP) becoming more distinct as the second TE increased.

The SNR and CNR measurements (Table 1) confirmed the above observations quantitatively. In the spin echo T1-w and T2-w images, the signal intensity of the NP was higher than that of either the CEP or the VEP, resulting in a CNR for CEP-NP of −4.1 (i.e., lower signal intensity of the CEP than of the NP) on average, and a CNR for the CEP-VEP of 2.2 in T1-w images and 0.0 in T2-w images, suggesting a low contrast between the CEP and the VEP in these sequences. In contrast, in the 3D Cones source images at the shortest TE of 0.032 ms, the SNR of the CEP was the highest (16.7) compared to the SNRs of the NP (15.0) and the VEP (10.4). This resulted in CNRs for the CEP-NP and CEP-VEP of 1.8 and 6.3, respectively, suggesting a moderately high contrast between the CEP and the VEP. These CNR values measured on the 3D Cones source images at TE = 0.032 ms were markedly different (*p* = 0.06 for CEP-NP; *p* = 0.001 for CEP-VEP; each with power of 0.99 or greater) from those from the spin echo T1-w and T2-w images. In the 3D Cones subtraction images at the second TE of 16 ms (i.e., first TE = 0.032 ms minus second TE = 16 ms), the CNR values were 4.5 and 2.5 for CEP-NP and CEP-VEP, respectively, suggesting a higher contrast between the CEP and the NP and a lower contrast between the CEP and the VEP.

**Figure 5:**
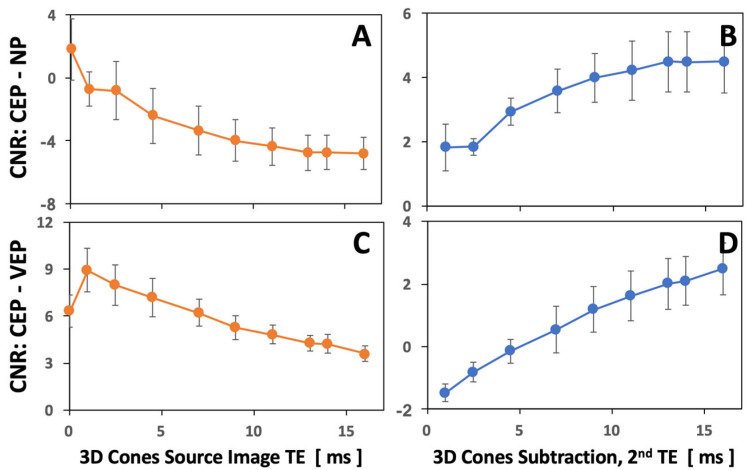
Contrast-to-noise ratio (CNR) of cartilage endplate (CEP) minus nucleus pulposus (NP) (**A**,**B**) and CEP minus vertebral endplate (VEP) (**C**,**D**) in the 3D Cones source images (**A**,**C**) and subtraction images (**B**,**D**), as a function of varying echo times (TEs).

In addition to Table 1, we created CNR-vs.-TE plots (Figure 5) to illustrate these findings on the source images (Figure 2A,C) and the subtraction images (Figure 2B,D). Using only the source images, it was difficult to achieve high CNR values for both CEP-NP and CEP-VEP. In contrast, using the subtraction images, increasing CNR values for both CEP-NP and CEP-VEP could be seen as the second TE was increased. For the subtraction images, statistically greater CNR values were found with second TE values greater than 10 ms (each *p* < 0.05, power > 0.94). The plots illustrate that different combinations of image contrasts were attainable with the different techniques (source images vs. subtraction images) as well as by varying the TEs.

## 4. Discussion

The present study aimed to investigate the optimal UTE MRI protocol, using 3D Cones sequences, for visualizing the tissues near the disco-vertebral junction including NP, CEP, and VEP. We utilized direct measurements of the signal intensity in both source images and echo subtractions images of human lumbar spines acquired using different TEs. Our findings provide valuable insights into optimizing the scan parameters for UTE MRI, enabling a better contrast between different intended spinal tissues.

The results demonstrated that the 3D Cones sequences, varying the TE spacing, could significantly impact image contrast, SNR, and CNR in both source images and subtraction images. Unlike the spin echo images that did not facilitate distinguishing the CEP from the VEP, the 3D Cones source images showed good contrast between the CEP and the VEP tissues at the shortest TEs. Additionally, in the 3D Cones subtraction images, increasing the second TE value increased the CNR between CEP and NP and between CEP and VEP, demonstrating that the image contrast at the DVJ can be modulated using different techniques as well as by varying the TEs.

There have been a few reports on the optimization of the MR imaging of the cartilage endplate. One notable study [25] utilizing fast low-angle shot pulse sequences performed an optimization of the T1 contrast between the NP and the CEP, using varying TRs and flip angles. The study found the optimal TR of 9 ms and flip angle of 20 degrees. However, this study did not investigate the use of echo subtraction or the effect of varying the TE. In another study [26], a 3D UTE sequence with TE values of 0.16 (shortest), 4.6, 9.2, 13.8, and 18.2 ms was used to acquire MR images of the lumbar spines of volunteers, and echo subtraction images were created to measure the CNR between the CEP and the VEP. This study also found the highest CNR when the second TEs of 13.8 and 18.4 ms were used, corroborating the results of the present study. In another study comparing four different UTE sequences with varying fat suppression schemes [27], CNR values ranging from 5 to 23 were determined, similar to those found in the non-subtracted source images in our study. Lastly, using a 3D UTE with radial acquisition and subtraction of two images acquired at TEs of 0.03 and 6.0 ms, Ji et al. [28] found a mean CNR between the CEP and the NP of about 9, slightly higher than the values found in our study.

There are several limitations in our study. One major issue is that the number of samples was small, due to the limited availability of cadaveric specimens at the time of the study. The small number of samples limits the generalizability of the results, unfortunately. However, this study presents an early technical development, and it is presumed that the signal intensity of normal CEP and VEP may not vary markedly between samples. For example, sample-to-sample variability calculated as the coefficient of variation (standard deviation divided by the mean) for the SNR of the CEP using various 3D UTE techniques averaged about 22%, suggesting a reasonably low variability. Nonetheless, a larger study will be needed in the future to confirm the present findings and to make the results generalizable. The use of only a single reader is another limitation, but our past study [17] showed a high inter-reader agreement when evaluating the DVJ on UTE MR images. Additionally, this study investigated only the effect of varying the TE, even though there are other scan parameters that could be adjusted, including the TR and the flip angle. The range of usable TR values in the 3D Cones sequences is narrow (roughly 20 to 100 ms), as longer TR will lengthen the scan duration. The flip angle is often set automatically for UTE sequences to maximize the SNR (i.e., Ernst angle) but can alter the image contrast. Additionally, while this work did not address it, there is a need to further optimize the UTE protocol to make it effective in clinical settings, including reducing the scan time, increasing patient comfort, etc. In vivo, the scan times for UTE MRI of the lumbar spine are still somewhat long, ranging from about 2 min for a single echo [27] to 3 min for a dual echo [28] and to as long as 10 min if inversion–recovery preparations are used [27]. Shorter scan times in the range of 1 to 2 min would be preferred for wider adaptation.

While not investigated in this study, an increasing number of studies on UTE MRI have suggested the importance of DVJ evaluation and the implications of an abnormal DVJ on spinal health. Our recent work in cadaveric spines [29] as well as in human subjects [28,30] found an association between lumbar spine DVJ abnormalities seen on UTE MRI and adjacent IVD degeneration [28,29,30]. In our study [29], an abnormal DVJ morphology including focal signal loss at the CEP as well as irregularity of the linear signal intensity of the CEP was found in about a third of all DVJs evaluated, and the frequency of abnormal DVJ increased with higher age and lower spinal level, e.g., L5 compared to L2 or L3. Additionally, discs adjacent to an abnormal DVJ had higher graders and lower T2 values of the NP. The nature of the CEP abnormality is currently unclear, but some authors speculated that it is related to ossification with aging [31] or calcification [32]. Future work on the DVJ can benefit from improved imaging, supported by studies to optimize the imaging protocols. Lastly, although the present work focused on optimizing the CNR for a normal DVJ, there is a need to evaluate many different types of lesions for different clinical purposes, for example to evaluate bone fractures [33] or longitudinal ligaments [34]. Additional work is needed to determine the optimal parameters for each intended purpose.

This study’s findings have important implications for clinical practice and research applications. By optimizing UTE MRI protocols to visualize the CEP more effectively, clinicians can better diagnose and monitor the MR changes at the disco-vertebral junction and better understand if and how the changes are related to low back pain and other pathologies of the lumbar spine. The natural history of DVJ abnormalities is a particular gap in knowledge that could be studied effectively with optimized UTE MRI protocols. Our results highlight the importance of considering the TE spacing when designing UTE MRI protocols for specific tissue contrast requirements. CNR-vs.-TE plots provide a useful tool for visualizing these relationships and selecting the optimal scan parameters.

## 5. Conclusions

In conclusion, our study highlights the feasibility of incorporating UTE MRI for the evaluation of the DVJ and its advantages over conventional spin echo sequences for improving the contrast between the CEP and adjacent tissues. Additionally, modulation of the contrast for the target tissues can be achieved using either source images or subtraction images, as well as by varying the echo times.

## Figures and Tables

**Table 1:** Signal-to-noise ratios (SNRs) for cartilage endplate (CEP), nucleus pulposus (NP), and vertebral endplate (VEP), as well as contrast-to-noise ratios (CNRs) for CEP minus NP and CEP minus VEP in images acquired using MR sequences of spin echo T1-weighted (SE T1), spin echo T2-weighted (SE T2), 3D Cones, and 3D Cones subtraction images.

			SNR			CNR	
Sequence	TR [ms]	TE [ms]	CEP	NP	VEP	CEP−NP	CEP−VEP
**SE T1-w**	650	10	12.4 (0.8)	16.5 (4.1)	10.2 (1.3)	−4.1 (4.2)	2.2 (1.8)
**SE T2-w**	3700	100	3.8 (1.0)	7.9 (2.6)	3.8 (0.9)	−4.1 (2.8)	0.0 (0.9)
**3D Cones**	50	0.03	16.7 (3.6)	15.0 (1.1)	10.4 (2.2)	1.8 (3.4)	6.3 (1.8)
	50	2.5	13.7 (3.2)	14.6 (0.8)	5.7 (1.6)	−0.8 (3.2)	8.0 (2.2)
	50	7	9.2 (2.1)	12.7 (0.8)	3.0 (0.8)	−3.4 (2.7)	6.2 (1.5)
	50	16	5.4 (0.9)	10.3 (0.9)	1.8 (0.2)	−4.8 (1.8)	3.6 (0.9)
**3D Cones Subtraction**	**TE 1 [ms]**	**TE 2 [ms]**					
	0.03	2.5	2.9 (0.7)	1.0 (0.4)	3.7 (1.1)	1.8 (0.5)	−0.8 (0.6)
	0.03	7	6.2 (1.5)	2.6 (0.6)	5.7 (1.3)	3.6 (1.2)	0.5 (1.3)
	0.03	16	9.2 (2.4)	4.7 (1.0)	6.7 (1.6)	4.5 (1.7)	2.5 (1.4)

## Data Availability

The data that support the findings of this study are not publicly available due to reasons of sensitivity. Anonymized data may be available from the corresponding author upon a review of the request. Data are located in controlled-access data storage at the corresponding author’s institution.
